# The Trigger in IVF Cycles: Molecular Pathways and Clinical Implications

**DOI:** 10.3390/ijms262411962

**Published:** 2025-12-11

**Authors:** Giorgio Maria Baldini, Domenico Baldini, Dario Lot, Daniele Ferri, Antonio Malvasi, Bernard Fioretti, Maria Matteo, Raoul Orvieto

**Affiliations:** 1 Obstetrics and Gynecology Unit, Department of Biomedical Sciences and Human Oncology, University of Bari “Aldo Moro”, 70121 Bari, Italy; gbaldini97@gmail.com (G.M.B.); antoniomalvasi@gmail.com (A.M.); 2 IVF Center, Momo Fertilife, 76011 Bisceglie, Italy; dariolot92@gmail.com (D.L.); danieleferrimomo@gmail.com (D.F.); 3 Department of Chemistry, Biology and Biotechnologies, University of Perugia, Via dell’ Elce di Sotto 8, 06132 Perugia, Italy; bernard.fioretti@unipg.it; 4 Department of Medical and Surgical Sciences, University of Foggia, 71122 Foggia, Italy; maria.matteo@unifg.it; 5 Department of Obstetrics and Gynecology, Chaim Sheba Medical Center, Tel HaShomer, Ramat Gan 5262000, Israel; raoul.orvieto@sheba.health.gov.il

**Keywords:** in vitro fertilization (IVF), oocyte maturation, hCG trigger, GnRH agonist, dual trigger, double trigger, kisspeptin

## Abstract

The final trigger of oocyte maturation is a pivotal step in assisted reproductive technology (ART). Different molecules and protocols—including human chorionic gonadotropin (hCG), gonadotropin-releasing hormone agonists (GnRHa), the dual trigger, the double trigger, and emerging agents such as kisspeptin—have been investigated to optimize oocyte competence, embryo development, and pregnancy outcomes while minimizing the risk of ovarian hyperstimulation syndrome (OHSS). HCG remains the most widely used trigger, but its pharmacological profile is associated with a significant risk of OHSS. GnRHa has emerged as an alternative in antagonist cycles, abolishing the risk of severe OHSS but often requiring tailored luteal phase support. Several strategies, including hCG, GnRHa, and combined approaches, have shown improvements in specific outcomes such as the oocyte maturity (MII) rate, fertilization rate, embryo development parameters, and, in selected contexts, a reduction in OHSS risk. Kisspeptin represents a promising option; however, its use remains predominantly within the research setting, with clinical application still limited to early-phase or highly selected studies. Beyond the choice of molecule, the timing of trigger administration—adjusted to follicle size, estradiol concentrations, and progesterone levels—also influences oocyte competence and subsequent clinical outcomes. Triggering final oocyte maturation remains a multifaceted decision that should be individualized according to patient characteristics, ovarian response, and risk of OHSS. Although hCG remains the historical reference standard, accumulating but heterogeneous evidence suggests that GnRHa-based strategies, including dual-trigger protocols, may improve specific outcomes in selected patient subgroups. However, results across trials are inconsistent, particularly in poor responders, and any exposure to hCG maintains a residual risk of OHSS. Kisspeptin represents a promising but still experimental option, with current data largely limited to early-phase clinical studies in highly selected high-risk populations. Well-designed randomized trials are required to clarify the true impact of these strategies on live birth, to refine timing and dosing, and to better define which patients are most likely to benefit.

## 1. Introduction

Final oocyte maturation represents a critical step in in vitro fertilization (IVF) protocols. Because the choice of the ovulation trigger directly influences the proportion of mature (MII) oocytes retrieved, fertilization rates, embryo development, and the risk of ovarian hyperstimulation syndrome (OHSS), it represents a key clinical decision in IVF cycles. The ovulation “trigger” is the event that induces cumulus expansion, meiotic resumption, luteinization, and subsequent ovulation. These events are orchestrated by the molecular cascade triggered by the midcycle LH surge, which coordinates the signaling pathways responsible for meiotic resumption and ovulation. This physiological framework underlies the rationale for the pharmacological agents currently used to induce final oocyte maturation in IVF. Over the years, various molecules and strategies have been developed to optimize the retrieval of mature oocytes, mitigate the risk of OHSS, and improve clinically relevant outcomes such as the number of mature oocytes, embryo quality, and pregnancy rates. This review aims to synthesize the current evidence on the pharmacological options and timing for the ovulatory trigger in IVF cycles, highlighting their advantages, limitations, and clinical implications.

## 2. Literature Search Methodology

This work was designed as a narrative review aiming to integrate physiological, pharmacological, and clinical evidence regarding final oocyte maturation in IVF cycles. A comprehensive literature search was conducted in PubMed, Scopus, and Embase, covering the period from 1990 to January 2025. The search strategy included the following terms, used individually or in combination: “IVF trigger,” “ovulation induction,” “hCG,” “GnRH agonist,” “dual trigger,” “double trigger,” “kisspeptin,” “oocyte maturation,” and “OHSS prevention”. Randomized controlled trials, prospective and retrospective cohort studies, meta-analyses, guideline documents, and expert consensus statements were considered eligible. Studies were included if they involved adult women undergoing IVF/ICSI cycles and reported outcomes related to oocyte maturation, embryo development, pregnancy, live birth, or OHSS. Case reports, editorials, letters, and animal studies were generally excluded, except when required to clarify underlying physiological mechanisms. Two authors independently screened titles and abstracts for relevance and subsequently assessed full texts. Any discrepancies were resolved through discussion with a third author. Due to the heterogeneity of study designs, populations, and outcome definitions, a quantitative synthesis (meta-analysis) was not feasible. This manuscript intentionally adopts a narrative approach. No PRISMA flow diagram was generated, no protocol was registered, and no formal assessment of risk of bias was performed. Given the substantial heterogeneity in study design, patient populations, stimulation protocols, trigger regimens, and outcome definitions, a formal meta-analysis was deemed inappropriate. Instead, we adopted a narrative synthesis with an explicit focus on the hierarchy of evidence. Where available, data from randomized controlled trials and meta-analyses were prioritized over observational or single-center retrospective studies, which were used primarily to generate hypotheses and to illustrate specific clinical scenarios. Because no formal risk-of-bias assessment was performed, all results should be interpreted with caution, and the strength of the conclusions is inherently limited by the quality and consistency of the underlying evidence. Table 1 offers an organized outline of the studies considered for this narrative review, showing the diversity of approaches, patient groups, and reported results encountered during the literature search. It gathers information from controlled trials, research reviews, and observational investigations, in accord with the preference for stronger scientific sources. The studies included explore a wide range of trigger methods applied in various clinical contexts. Since no formal evaluation of methodological quality was performed and the material is highly variable, the table is intended to function only as a descriptive summary of the available publications. Its role is to support the broader discussion of physiological, pharmacological, and clinical aspects presented in the review.

## 3. Trigger Timing in IVF Cycles

### 3.1. Follicular Size and Trigger Timing

The correct timing of the ovulation trigger administration in in vitro fertilization (IVF) cycles is a crucial element for optimizing the retrieval of mature oocytes. Among the primary factors influencing the decision on when to trigger, follicular size plays a central role, as oocyte maturation is closely correlated with follicular development. Oocyte maturation and the capacity to resume meiosis are strictly linked to the follicle’s dimensions at the time of retrieval. Several studies have highlighted a significant relationship between oocyte diameter and its ability to complete maturation [18]. It has been demonstrated that oocytes with a diameter between 106 and 125 microns have a significantly higher probability of maturing compared to those of smaller dimensions (86–105 microns) [18]. Even in unstimulated conditions, oocyte size is a determining factor in the progression of meiotic maturation. The correlation between follicular size and oocyte development has been analyzed in the context of controlled ovarian stimulation [19]. A total of 640 follicles were evaluated, divided into three groups: large (≥16 mm), medium (13–15 mm), and small (<13 mm). The results showed a significantly higher oocyte retrieval rate in larger follicles (≥16 mm) (76.3% in large vs. 55.6% in small; *p* = 0.001), as well as a higher rate of mature (metaphase II) oocytes in large and medium follicles compared to small ones (*p* = 0.001 and *p* = 0.01, respectively). However, no significant differences were found in fertilization rates or embryo quality among mature oocytes, regardless of the size of the follicle of origin. These findings were confirm by others studies [20,21,22], which have shown that the oocyte maturation process is regulated not only by follicle size but also by the intrafollicular environment, including the presence of growth factors and regulatory molecules in the follicular fluid. Some studies [23,24] have demonstrated that there is a dimensional threshold above which the probability of obtaining mature (metaphase II) oocytes significantly increases. Traditionally, the trigger is administered when dominant follicles reach an average diameter between 16 and 22 mm [3]. However, recent research suggests that greater personalization based on the patient’s individual characteristics could improve clinical outcomes [3]. Indeed, the probability of retrieving mature oocytes increases with follicular growth, but not in a linear fashion [3]. It appears that follicles with a diameter of at least 18 mm have a significantly higher probability of containing mature oocytes compared to smaller ones [3]. Conversely, some studies suggest that retrieving oocytes from overly large follicles (>22–24 mm) may be associated with increased oocyte post-maturity and reduced embryo quality [25]. The relationship between follicular size and embryonic development potential was also evaluated [26], demonstrating that oocytes retrieved from follicles with a diameter between 17 and 22 mm have the highest fertilization and blastulation rates. In contrast, follicles smaller than 14 mm show a very low probability of containing mature oocytes, unless a double trigger was administered, suggesting that an early trigger may compromise oocyte quality and IVF outcomes. Based on the available evidence, the clinical strategy to optimize the number of obtainable mature oocytes consists of careful monitoring of follicular growth through serial ultrasound scans and measurements of serum estradiol levels. Although the commonly accepted threshold for trigger administration is the presence of dominant follicles ≥16 mm, some authors suggest that oocyte maturation can be optimized by considering not only follicular size but also the stimulation method and the patient’s individual characteristics [23]. In GnRH antagonist protocols, a GnRH agonist trigger is often administered when at least three follicles reach 17 mm to reduce the risk of Ovarian Hyperstimulation Syndrome (OHSS) while maintaining a good oocyte maturation rate [24,27]. A study of 492 cycles evaluated the ratio of mature to growing follicles on the day of the trigger. It was observed that patients with a mature follicle percentage of >60% achieve the best implantation and clinical pregnancy rates [5]. This finding supports the idea that the synchronization of follicular maturation is a key element in determining the success of IVF.

### 3.2. Correlation Between the Trigger and Endocrine Monitoring in Stimulated Cycles

**Figure 1:**
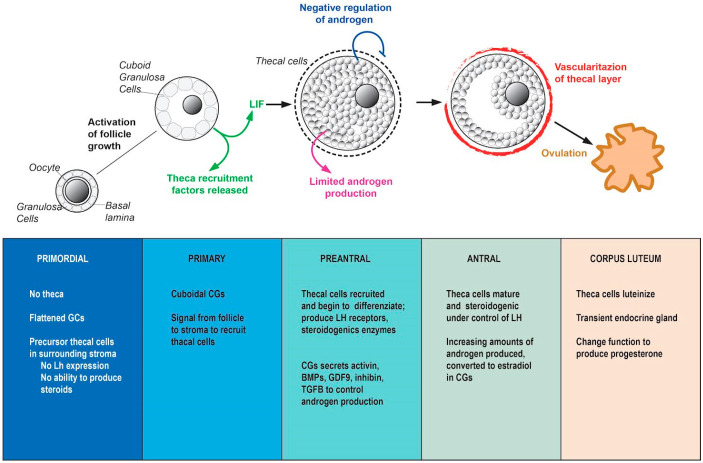
Hormonal pathways regulating human oocyte maturation.

Endocrine monitoring during ovarian stimulation is also essential for optimizing the timing of the ovulation trigger and maximizing the retrieval of mature oocytes in in vitro fertilization (IVF) cycles. Monitoring the levels of estradiol (E2), luteinizing hormone (LH), and progesterone (P4) provides crucial information about the follicular response and helps prevent complications such as premature luteinization, suboptimal response to GnRH agonist trigger and Ovarian Hyperstimulation Syndrome (OHSS) (Figure 1). One of the fundamental aspects is the relationship between serum estradiol levels and follicular size. Estradiol, produced by granulosa cells, indirectly reflects follicular maturity and the adequacy of stimulation. It has been shown that estradiol levels above a critical threshold (>3000–4000 pg/mL) can be associated with an increased risk of OHSS, making E2 monitoring essential for personalizing trigger administration [26]. The choice of the optimal timing of trigger often depends on follicular size and serum estradiol (E2) levels. An analysis of 606 natural cycle IVF cycles revealed that an estradiol level between 637 and 1480 pmol/L correlates with the LH surge and higher oocyte maturation rates [28]. The average follicular growth and estradiol increase were 1.04 mm and 167 pmol/L per day, respectively. The LH surge began at estradiol levels of 637 pmol/L in 25% of cases, 911 pmol/L in 50%, and 1480 pmol/L in 75% [28]. It has been evaluated how the decline in estradiol levels between the trigger day and the day of embryo transfer (ET) can influence the success of assisted reproduction [29]. Patients were divided into four groups based on the reduction in estradiol levels between the trigger day and the transfer day: <20% reduction; 20–40% reduction; 41–60% reduction; >60% reduction. The results showed that in patients with an estradiol level reduction of >60%, the clinical pregnancy and live birth rates were significantly lower, while the risk of spontaneous abortion was higher [29]. These findings suggest that monitoring estradiol levels after the trigger might help predicting cycle outcomes and optimize treatment management. LH monitoring is equally relevant, especially in GnRH antagonist protocols. Endogenous LH stimulates the final maturation of oocytes, but in stimulation protocols, its regulation is crucial to prevent premature ovulation. Orvieto et al. have suggested that the optimal pregnancy rates in patients undergoing the multiple GnRH antagonist COH protocol were observed in patients achieving, on day of hCG administration, an E2-to-follicle ratio < 100 pg/mL [30]. An endocrine parameter of growing interest is progesterone. A premature rise in progesterone (>1.5 ng/mL) before the trigger has been associated with a reduction in endometrial receptivity, with negative effects on embryo implantation rates [31]. The impact of the progesterone peak on embryo development kinetics was recently analyzed, demonstrating that high P4 levels on the trigger day do not influence embryo morphokinetics, suggesting that endocrine alterations during stimulation may have direct repercussions on endometrial quality [31]. Furthermore, it had already been shown that elevated progesterone levels at the time of hCG administration do not affect embryo quality and reproductive outcomes in frozen embryo transfer cycles [32].

### 3.3. Specific Considerations on Timing

The effects of delaying hCG administration by 24 h in cycles with low progesterone levels (<1 ng/mL) have been studied. Although some studies found no significant differences in fertilization and implantation rates [33], others have reported an increase in the number of mature oocytes in groups where the trigger was postponed [34]. Therefore, delaying the trigger may represent a beneficial strategy only in specific patient subgroups. For patients over 40 years of age, early triggering in the GnRH antagonist protocol has proven advantageous in terms of reducing the duration of stimulation and increasing the number of high-quality embryos. However, live birth rates were not significantly different compared to patients treated with standard timing [35]. This suggests that an early trigger might improve certain aspects of the treatment without necessarily increasing overall success rates. Age is a determining factor in the success of assisted reproduction. The same study analyzed the effect of early triggering in 1529 patients across different age groups. For patients under 35 years old, early triggering reduced the number of retrieved oocytes without improving pregnancy rates. In the 35–40 age group, no significant difference was observed between early and standard triggering. However, for patients over 40, early triggering increased the number of high-quality embryos and improved the clinical pregnancy rate [35]. These results suggest that in patients over 40, an early trigger could improve the chances of success.

### 3.4. Timing of Oocyte Pick-Up

One of the most studied aspects in the timing of oocyte retrieval is the time interval between the ovulation trigger and the oocyte pick-up. Traditionally, ovulation is induced with human chorionic gonadotropin (hCG) or a GnRH agonist, and the retrieval is performed approximately 34–37 h later. However, some authors suggest that a longer or shorter interval could influence the number of mature oocytes retrieved and the fertilization rate [35]. A study that analyzed 438 IVF cycles compared two groups: those with a trigger-to-retrieval interval of <36 h had an average of 11.86 oocytes retrieved per cycle, while a group with a trigger-to-retrieval interval of ≥36 h had an average of 12.24 oocytes retrieved per cycle. No statistically significant difference was found between the two groups, suggesting that a variation in a few hours in the pick-up time does not significantly affect oocyte maturation [36]. Another study also confirmed that the timing of the pick-up can be flexible and that an interval between 32 and 39 h does not compromise clinical outcomes. However, in specific cases of high ovarian response, it may be necessary to advance the pick-up to avoid the risk of spontaneous ovulation and to reduce the risk of Ovarian Hyperstimulation Syndrome (OHSS) [37].

### 3.5. The Role of Molecules Assisting the Trigger

The ovulation trigger in controlled ovarian stimulation (COH) cycles for in vitro fertilization (IVF) is a highly regulated process involving a series of hormones, growth factors, and intracellular signals. Oocyte maturation is a key event in IVF success and depends on the synchronization between follicular growth and the activation of specific signaling pathways. Among the molecules involved in this process are the gonadotropins (FSH and LH), epidermal growth factor (EGF), growth hormone (GH), inhibins, insulin-like growth factor (IGF), and various antioxidants. Understanding their roles and interactions can help improve stimulation protocols and maximize pregnancy rates in assisted reproductive treatments. FSH (follicle-stimulating hormone) and LH (luteinizing hormone) are two key hormones in the regulation of folliculogenesis and oocyte maturation. Cadenas et al. [38] highlighted that FSH and LH play a crucial role in the resumption of meiosis and the progression of oocyte maturation to metaphase II (Figure 1). They demonstrated that oocytes exposed to physiological levels of these gonadotropins show a significantly higher percentage of maturation completion compared to those not exposed. Furthermore, they suggested that the increase in FSH within the follicle coincides with the generation of an essential positive signal required to complete oocyte maturation [38], by upregulating the LH receptor, which correlated with MII transition.

**Figure 2:**
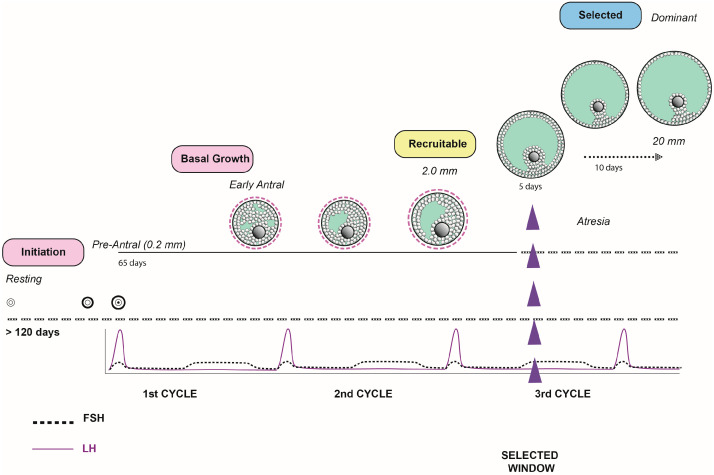
LH surge kinetics.

The impact of LH on oocyte quality in primates has been the subject of further research [39]. The results demonstrated that the amplitude and duration of the LH surge directly influence the quality of the matured oocyte. The authors observed that an hCG injection induces a sustained increase in LH bioactivity for over 72 h, whereas induction with GnRH agonists generates a more transient surge. This difference in LH kinetics is reflected in the proportion of oocytes that reach metaphase II, suggesting that the temporal regulation of LH is a determinant of oocyte quality [39] (Figure 2).

Arroyo et al. (2020) [40] identified the main signaling pathways activated by LH in human oocyte maturation. Specifically, the authors demonstrated that the CNP/NPR2 system and the network of epidermal growth factor-like factors (EGF-like factors) play an essential role in the transduction of the ovulatory signal. The activation of these pathways not only facilitates meiotic resumption but is also correlated with subsequent embryo quality [40]. EGF and its ligands (amphiregulin, epiregulin, and betacellulin) are essential for oocyte maturation and the acquisition of embryonic competence. The EGF signal is activated by LH and transmitted from the granulosa cells to the oocyte through gap junctions, promoting meiotic resumption. It has been shown [38] that incubating oocytes with EGF and IGF-I significantly increases the percentage of oocytes reaching metaphase II, suggesting that these growth factors play a key role in regulating oocyte maturation [38]. Furthermore, it has been highlighted that estradiol, through the activation of GPER, stimulates the production of EGF-like factors in cumulus cells, promoting oocyte maturation and improving embryo quality [41]. Growth hormone (GH) has also been studied for its potential role in enhancing the ovarian response to gonadotropin stimulation and promoting oocyte maturation. However, the results are still conflicting, and further randomized controlled trials are needed to establish the definitive efficacy of GH in clinical practice [38]. Inhibins are glycoproteins produced by granulosa cells that play a key role in regulating ovarian function. Inhibin A and inhibin B are involved in the negative feedback regulation of FSH and can influence oocyte quality and embryo development. Inhibin A and activin A act on oocyte maturation in primates [42]; the addition of these molecules to in vitro culture media significantly increased the percentage of oocytes reaching metaphase II and the fertilization rate. The concentration of inhibin B in follicular fluid and its correlation with embryo quality was the subject of another study [43]. The results showed that higher levels of inhibin B in follicular fluid are associated with better embryo development and a higher probability of implantation. Moreover, the authors highlighted that inhibin B decreases with age, reflecting a decline in ovarian reserve and oocyte quality. Lawrenz et al. [44] proposed inhibin A as a biomarker to determine the optimal moment for the ovulation trigger in ovarian stimulation cycles. The results indicated that inhibin A levels are strongly correlated with the number of mature follicles (>15 mm) and the number of retrieved oocytes, suggesting that this hormone may represent a more reliable indicator than estradiol for establishing the optimal trigger timing. Antioxidants may play an essential role in maintaining oocyte quality by protecting cells from oxidative stress induced by reactive oxygen species (ROS). The LH surge preceding ovulation induces a massive production of ROS, which are necessary to modulate fundamental reproductive functions, including oocyte maturation, ovarian steroidogenesis, and luteolysis [45]. However, an imbalance between ROS production and antioxidant defense mechanisms can compromise oocyte quality, alter mitochondrial function, and reduce fertilization and embryo development rates. At present, however, these assessments require confirmation from large-scale and prospective studies.

## 4. Trigger Molecules

### 4.1. Kisspeptin

One of the key aspects in the study of kisspeptin and its role in oocyte maturation is its expression and function within the follicular microenvironment. The role of kisspeptin signaling in oocyte maturation has been investigated both centrally and at the ovarian level [46]. A study conducted by Kuspinar et al. [47] analyzed the expression levels of the kisspeptin gene (KISS1) and its receptor (KISS1R) in cumulus cells (CCs) and follicular fluid (FF) of patients undergoing oocyte retrieval for IVF. Oocytes were divided into three groups according to their nuclear maturation and fertilization status: oocytes in metaphase I or germinal vesicle stage (incomplete nuclear maturation); unfertilized metaphase II oocytes (incomplete cytoplasmic maturation); and fertilized metaphase II oocytes (complete nuclear and cytoplasmic maturation). Gene expression levels were assessed by RT-PCR, while protein levels of KISS1 and KISS1R were measured by ELISA. No significant differences were observed among the groups. In particular, kisspeptin levels in cumulus cells and follicular fluid were not correlated with either nuclear or cytoplasmic maturation of the oocyte. The action of kisspeptin on oocyte maturation is thought to be primarily mediated by its stimulation of gonadotropin release through modulation of hypothalamic GnRH-producing neurons. On the other hand, Kisspeptin and its receptor are expressed within the ovary, suggesting a direct effect on oocyte maturation. Pituitary-derived gonadotropins stimulate kisspeptin expression in granulosa cells (GCs). Ovarian kisspeptin levels increase progressively during the estrogenic phase, reaching a peak before the pre-ovulatory LH surge. In mice lacking the KISS1R receptor in oocytes, maturation and ovulation fail to occur, supporting an essential role for kisspeptin in these processes [46]. These results support the experimental hypothesis that kisspeptin may act directly on oocytes through KISS1R receptors, promoting maturation in response to gonadotropic signaling. Sharma et al. [48] evaluated the potential of kisspeptin to improve the safety and efficacy of ovarian stimulation. Currently, hCG triggering is associated with a high risk of OHSS, as it induces a prolonged LH effect leading to excessive estradiol secretion and increased production of vascular permeability factors. GnRH agonists can mitigate this risk but are associated with impaired luteal function and lower implantation rates. By stimulating endogenous GnRH release in a physiological manner, kisspeptin may represent a potentially safe alternative for ovulation triggering, although current evidence derives mainly from early-phase clinical studies. Clinical studies conducted to date [15,16] have demonstrated that a single injection of kisspeptin-54 is sufficient to induce an LH surge adequate to complete oocyte maturation in the majority of patients. Preliminary data suggest efficacy similar to GnRH agonists, although direct comparative trials are limited. These findings indicate that kisspeptin represents an interesting emerging option, with a favorable safety profile in early studies, though its impact on IVF success rates requires validation in larger randomized trials. One of the most significant studies on the use of kisspeptin as an ovulation trigger was conducted by Abbara et al. [15], who evaluated the efficacy and safety of kisspeptin-54 in women at high risk of developing OHSS during IVF treatment. Oocyte maturation was achieved in 95% of women following kisspeptin administration. The highest oocyte maturation rate was observed with the 12.8 nmol/kg dose, with a 69% increase compared to the lowest dose tested. Biochemical pregnancy, clinical pregnancy, and live birth rates per embryo transfer were 63%, 53%, and 45%, respectively. No cases of moderate, severe, or critical OHSS were reported. These findings suggest induce effective and safe ovulation in highly selected high-risk patients, although confirmation in larger and more diverse cohorts is needed. The same author [16] explored whether oocyte maturation could be further improved through a second administration of kisspeptin-54, given 10 h after the initial injection. Patients were randomized into two groups: a single-dose group, receiving only the first injection, and a double-dose group, receiving a second kisspeptin-54 injection after 10 h. In the double-dose group, 71% of patients achieved an oocyte yield ≥60%, compared to 45% in the single-dose group (*p* = 0.042). The second injection enhanced LH secretion, improving oocyte yield. No cases of moderate or severe OHSS were recorded, confirming the favorable safety profile of kisspeptin. These preliminary findings indicate that a double-dose regimen may further improve oocyte maturation, but require replication in larger randomized trials. Follicular kisspeptin concentrations in IVF patients have also been studied [49]. Follicular kisspeptin levels were significantly higher than plasma concentrations and were positively correlated with estradiol levels and the number of mature oocytes retrieved. Peak plasma kisspeptin levels were observed on the day of oocyte retrieval and embryo transfer. These findings suggest that kisspeptin may play a physiological role in supporting oocyte maturation and ovulation, in addition to its central role in regulating the hypothalamic-pituitary-gonadal (HPG) axis although causal mechanisms remain to be fully elucidated. Kisspeptin has been compared with other ovulation-triggering agents to assess its advantages and limitations. It mimics the LH surge, directly stimulating oocyte maturation; it induces endogenous LH release from the pituitary; it carries a lower OHSS risk compared to hCG; yet, it requires stronger luteal support and may be associated with reduced implantation rates. Kisspeptin-54 has demonstrated comparable efficacy to hCG and GnRHa in promoting oocyte maturation [50], with the additional benefit of a superior safety profile in women at risk of OHSS. Nevertheless, several questions remain unanswered: What is the optimal dosing regimen to maximize pregnancy rates while maintaining safety? What are the long-term effects of kisspeptin use in fertility treatments? Which subgroups of patients may benefit most from this approach? Further large-scale randomized clinical trials will be required to address these issues and to establish kisspeptin as a standard option in controlled ovarian stimulation protocols.

### 4.2. hCG

In conventional controlled ovarian hyperstimulation (COH) protocols for IVF-ICSI, final follicular maturation is usually triggered by the administration of a bolus of human chorionic gonadotropin (hCG; 5000–10,000 IU) approximately 36 h prior to oocyte retrieval. hCG plays a pivotal role in inducing the resumption of oocyte meiosis, cumulus expansion, and granulosa cell luteinization. Given its central role, the optimal dosing and mode of administration of hCG have been extensively investigated [24,25,26,30,51,52,53,54] in an effort to maximize clinical outcomes while minimizing the risk of ovarian hyperstimulation syndrome (OHSS). OHSS typically occurs either 3–7 days following hCG administration in high-risk patients (early-onset OHSS), or during early pregnancy, 12–17 days after administration (late-onset OHSS). Tailoring the ovulation trigger to patient-specific risk factors and ovarian response, combined with the use of a freeze-all strategy, can substantially reduce the incidence and severity of OHSS. Completely avoiding hCG as a trigger effectively eliminates the risk of severe early-onset OHSS, but at the expense of higher treatment costs, longer treatment duration, and potentially greater patient burden. Due to its structural similarity to luteinizing hormone (LH), hCG binds to LH receptors on granulosa cells, thereby mimicking the physiological LH surge. However, compared with endogenous LH, hCG has a significantly longer half-life, which ensures prolonged luteal support but also increases the risk of OHSS in predisposed patients. One of the most debated aspects of hCG triggering concerns the optimal dosage. A prospective randomized trial by Singh et al. [51], involving 100 women aged 21–40 years with normal or diminished ovarian reserve, compared 250 µg versus 500 µg of hCG. While the mean number of retrieved oocytes was comparable between groups, the number of mature oocytes and fertilization rates were significantly higher with 500 µg, particularly in poor responders. However, pregnancy rates did not differ between groups. Similarly, the effect of reduced hCG dosing was explored in women with PCOS undergoing stimulation with recombinant FSH and GnRH antagonists [52]. Administration of 10,000, 5000, or 2500 IU hCG yielded comparable fertilization and pregnancy rates, suggesting that lower doses may mitigate OHSS risk compromising fertilization, embryo quality, or pregnancy outcomes. Although the number of retrieved oocytes, MII oocytes, and fertilization rates was lower in the 2500 IU group, embryo quality and ongoing pregnancy rates were similar, with a tendency toward improved implantation outcomes at the lowest dose. Other studies evaluated the impact of BMI on the efficacy of hCG triggering. Lin et al. [53] compared two HCG trgger doses (4000 IU versus 6000 IU) in IVF/ICSI cycles. A dose of 6000 IU significantly improved pregnancy rates among women with a BMI between 20 and 25 kg/m^2^, although the number of mature oocytes retrieved did not differ substantially. On the other hand, Ashkenazi et al. [54] compared the number and percent of M-II and cycle outcome in women triggered with 10,000 IU of hCG whose were in the 90 th BMI percentile to those in the 10 th percentile. No differences were observed between the groups in mean patients’ age, number of gonadotropin ampoules used, mean number of oocytes retrieved or the number and percentage of mature M-II oocytes. This may imply that the 10,000 IU hCG dosage is much higher than the dosage that is actually required. The endocrine consequences of different hCG dosages during the luteal phase were investigated in a randomized controlled trial by Svenstrup et al. [55]. The study demonstrated that higher doses of hCG (5000, 6500, and 10,000 IU) were associated with increased endogenous progesterone production, suggesting that the dose of hCG administered can modulate luteal phase hormonal dynamics and potentially influence implantation success.

### 4.3. Alternative Approaches and Limitations of hCG

The introduction of recombinant molecules such as recombinant hCG (rhCG) and recombinant LH (rLH) has enabled a more physiological mimicry of the endogenous LH surge. A meta-analysis [56] including 14 randomized trials with a total of 2306 participants found no significant differences between rhCG and urinary hCG (uhCG) in terms of ongoing pregnancy or live birth rates, nor in the risk of OHSS. Similarly, rLH did not demonstrate substantial differences compared with rhCG. Nevertheless, Andersen et al. [57] reported that administration of a bolus of hCG (5000–10,000 IU) induces a non-physiological luteal endocrine profile, characterized by a more rapid and pronounced rise in progesterone levels compared to natural cycles, leading to accelerated endometrial development that may be detrimental to implantation. In addition, a large retrospective study analyzing more than 8000 IVF/ICSI cycles demonstrated that serum progesterone levels ≥1.75 ng/mL at the time of hCG triggering were significantly associated with reduced clinical pregnancy and live birth rates, thereby supporting the adoption of elective embryo cryopreservation strategies in such cases [17]. Despite these limitations, hCG remains the standard pharmacological agent for inducing final oocyte maturation in assisted reproduction cycles.

### 4.4. GnRH Analog as a Trigger

In GnRH antagonist (GnRH-ant) protocols, the use of a GnRH agonist (GnRHa) as an alternative to hCG for final oocyte maturation has been proposed to reduce the risk of OHSS. However, this strategy has been associated with lower implantation and clinical pregnancy rates, as well as higher early pregnancy loss. Kol and Humaidan [58] proposed three possible approaches to optimize outcomes: the freeze-all strategy, fresh embryo transfer with intensive luteal phase support, and fresh embryo transfer with low-dose hCG supplementation. Subsequently, Orvieto et al. [59] evaluated a protocol combining GnRHa trigger with antagonist co-treatment, showing that the dual trigger (GnRHa plus low-dose hCG) can improve luteal function while maintaining a reduced risk of OHSS in high-risk patients. The use of GnRHa for final oocyte maturation has become a cornerstone of GnRH-ant protocols and is also considered safe with respect to obstetric and neonatal outcomes. Budinetz et al. [60] found no significant differences in congenital anomalies or maternal/neonatal complications between GnRHa and hCG triggers. Since pituitary desensitization does not occur in antagonist cycles, gonadotrophs remain responsive to GnRH. When used as a trigger, GnRHa replaces the antagonist at the pituitary receptor, inducing an endogenous surge of both LH and FSH [61]. Compared with the physiological LH surge, the GnRHa-induced surge is shorter (ascending phase ~4 h, descending phase ~20 h, versus ~48 h in a natural cycle), leading to early luteolysis and effective prevention of OHSS [2]. The main clinical advantage of the GnRHa trigger is therefore the marked reduction in OHSS risk, mediated in part by reduced VEGF secretion. During the mid-luteal phase, ovarian volume and pelvic free fluid are significantly lower compared with hCG cycles [2,62,63]. Evidence from prospective, retrospective, and randomized studies in high responders and oocyte donors shows near-complete elimination of OHSS with GnRHa [2,64,65,66]. In women with PCOS, one RCT reported no cases of moderate/severe OHSS with GnRHa trigger compared with a 31% incidence with hCG trigger and fresh transfer [64]. Observational studies have also confirmed the absence of OHSS in extremely high responders [65,66]. Rare cases of severe OHSS have been reported following GnRHa trigger without hCG exposure; in such instances, activating FSH receptor mutations should be considered, as well as accidental administration or endogenous hCG secretion (pregnancy, including ectopic, or paraneoplastic syndromes) [67,68,69]. Rapid luteolysis following GnRHa, if not managed properly, results in a luteal phase defect with reduced clinical pregnancy and live birth rates in fresh transfers. Effective strategies include cycle segmentation (freeze-all) or intensive luteal support, both of which have been well described in observational studies and systematic reviews [70,71,72]. Various dosing regimens have been employed, often without true dose-finding studies. For leuprolide, single doses ranging from 0.5 to 4 mg and even double injections 12 h apart have been reported; a single 1 mg dose appears effective for optimal MII yield [64,73,74,75,76,77,78,79,80]. Triptorelin has been used more consistently at 0.2 mg in most studies, and a dose-finding RCT in oocyte donors (0.2/0.3/0.4 mg) reported no significant differences in MII yield, fertilization, embryo development, or clinical outcomes [1,81,82,83,84,85]. For buserelin, dose-finding studies suggest a minimum effective dose, and both intranasal (50 µg) and subcutaneous (2 mg) regimens have achieved good outcomes [86,87,88,89,90]. Predictors of oocyte yield following GnRHa trigger include serum LH and progesterone 8–12 h post-trigger, which correlate with both total and MII oocyte numbers. All cases of empty follicle syndrome (EFS) after GnRHa trigger were associated with LH <15 IU/L and progesterone <3.5 ng/mL. Other indicators of suboptimal response to GnRH agonist trigger are patients with irregular menses and being on long-term oral contraception and those with LH levels < 0.5 IU at the initiation of ovarian stimulation and at the trigger day [91]. In the absence of a hormonal rise, re-triggering with small dose (1000–1500 IU) of hCG followed by oocyte retrieval 34–36 h later may rescue the cycle, provided OHSS risk is acceptable [91]. In clinical practice, GnRHa triggering is typically performed 8–12 h after the last antagonist dose. Moreover, while a retrospective analysis (53 cycles) found no significant association between the interval from antagonist to agonist (mean 4.6 ± 2.7 h) and oocyte retrieval, maturation, or outcomes after adjustment for age and BMI [91], Another retrospective cohort study including 413 patients undergoing GnRH antagonist cycles in which GnRHagonist trigger was used, has demonstrated that while LH concentrations post-GnRHa trigger differ in regard to antagonist–agonist intervals (12–14 h; 7–10 h; 5–6 h and 2–4 h interval), the follicle/mature oocyte ratio achieved was not affected [92]. Recent meta-analyses suggest that discontinuing the antagonist on the day of trigger may increase the proportion of mature oocytes without increasing the risk of premature ovulation [62]. The luteal phase after GnRHa trigger is generally shorter (~9 days) compared with hCG (~13 days), likely due to the shorter LH surge, partial pituitary desensitization from the flare, and supraphysiological E2/P4 levels suppressing endogenous LH [72,93,94]. While GnRH-ant protocols effectively prevent OHSS, they may be associated with a modest reduction in oocyte yield (~1.5 fewer oocytes per cycle compared with long-agonist protocols) [72,95]. A Cochrane review reported that fresh autologous cycles with GnRHa trigger had lower live birth and ongoing pregnancy rates and higher early miscarriage rates compared with hCG [1]. This disadvantage is attributed to suppression of endogenous LH with cetrorelix/ganirelix and the shortened luteal window [73,94]. Freeze-all after GnRHa trigger is the most widely used strategy. A meta-analysis reported higher live birth rates in eFET cycles compared with fresh transfers in high responders (RR 1.16; 95% CI 1.05–1.28), along with a significantly reduced risk of moderate/severe OHSS (RR 0.42; 95% CI 0.19–0.96) [96]. An RCT comparing freeze-all versus fresh transfer with GnRHa plus low-dose hCG luteal support showed clinically relevant OHSS only in the fresh transfer group, with similar pregnancy rates [97]. Intensive luteal supplementation with estradiol and progesterone can achieve outcomes comparable to hCG triggering in high responders: in one RCT including PCOS/high responders, monitored and “on-demand” adjusted supplementation yielded ongoing pregnancy rates of 53% versus 48.3% (GnRHa + intensive vs. hCG + standard) [63]. A prospective study employing repeated doses of GnRHa for luteal support reported no OHSS or adverse events, with a clinical pregnancy rate of 43.6% [98]. A SWOT analysis has summarized the strengths of GnRHa trigger (near-elimination of OHSS < 0.1% in at-risk populations, reduced patient discomfort due to smaller luteal volume, decreased VEGF levels) and its limitations (requirement for intensive luteal monitoring, risk of EFS in patients with impaired pituitary reserve, uncertainty regarding optimal dosing) [72]. Comparative studies have shown mixed results for intensive “luteal rescue” strategies compared with hCG, with some reporting lower implantation and pregnancy rates despite aggressive support [99,100].

### 4.5. Dual Trigger (GnRHa + hCG) and Double Trigger (Staggered GnRHa and hCG)

An innovative approach was introduced by Shapiro et al. [101], who first proposed the concept of the “dual trigger.” This strategy may be particularly advantageous for patients exhibiting suboptimal ovarian response or poor oocyte quality. The dual trigger, based on the co-administration of a GnRH agonist (GnRHa) and hCG, was designed to optimize final oocyte maturation in specific subgroups undergoing assisted reproductive technologies (ART). GnRHa induces an endogenous combined surge of LH and FSH, whereas hCG exerts prolonged luteotrophic activity through activation of the LH/CG receptor. The combination aims to exploit complementary signaling pathways (including activation of the AKT/ERK cascades and increased cAMP production), thereby enhancing meiotic resumption, cumulus–oocyte complex expansion, and luteinization, ultimately improving the yield of mature (MII) oocytes and embryo quality [9,101,102,103,104]. Available evidence from retrospective, prospective, and randomized controlled studies suggests that the dual trigger may increase the number of MII oocytes and expand the pool of cryopreservable embryos compared with hCG alone in selected populations—particularly normo-responders with a high proportion of immature oocytes, suboptimal response, or some categories of poor ovarian responders (POR) [9,10,103,104]. In one RCT involving normo-responders, simultaneous administration of GnRHa and hCG approximately 36 h before oocyte retrieval was associated with a higher probability of obtaining top-quality embryos and a greater number of cryopreserved embryos, although overall clinical outcomes were comparable to the hCG-only group [37]. Observational data also indicate benefits in scenarios characterized by a high prevalence of immature oocytes, despite the possibility that intrinsic oocyte dysfunction may limit improvements in key reproductive outcomes such as pregnancy and live birth [9,103,104]. In the context of GnRHa triggering, luteal rescue with 1500 IU of hCG has been proposed, administered either close to oocyte retrieval (3 days, 35 h post-trigger, or 1 h after retrieval or according to alternative deferred schedules). These regimens can restore adequate luteal support and yield reproductive outcomes comparable to hCG triggering in selected groups [11]. However, the use of hCG does not eliminate OHSS risk, which must still be considered, particularly in high responders [11,105,106]. This underscores the importance of careful patient selection and informed counseling regarding the benefit–risk balance. The so-called “double trigger” involves non-simultaneous administration of the two agents (e.g., GnRHa ~40 h and hCG ~34 h prior to oocyte retrieval), aiming to extend the maturation window and maximize the effect of the endogenous FSH surge elicited by GnRHa. This strategy has been reported as useful in patients with a history of a high proportion of immature oocytes or those experiencing empty follicle syndrome (EFS) and low oocyte recovery rate [104,105,106,107]. Prospective studies indicate that double trigger protocols may increase MII oocyte yield, fertilization (2PN) rates, and the number of top-quality embryos in these subgroups, with an acceptable safety profile when hCG exposure is carefully modulated [9,10,11,104,107]. In clinical practice, dual and double trigger strategies are mainly considered in women with a high proportion of immature oocytes, selected poor responders, and patients with documented empty follicle syndrome or previous cycles with inadequate maturation despite appropriate follicular development. Dual trigger refers to the simultaneous administration of GnRHa and hCG approximately 34–36 h before oocyte retrieval, whereas double trigger denotes a staggered regimen (GnRHa around 40 h and hCG around 34 h before retrieval) designed to prolong the maturation window. Both approaches may increase the number of MII oocytes and cryopreservable embryos in these subgroups, but the magnitude and consistency of benefit vary across studies. Luteal rescue with low-dose hCG should be restricted to women at low-to-moderate risk of OHSS, bearing in mind that any hCG exposure preserves a residual, dose-dependent risk of OHSS, especially in high responders [4,9,10,11,63,103,104,107,108,109].

## 5. Comparison Between Molecules

The comparison of different ovulation trigger strategies for final oocyte maturation in IVF-ICSI cycles has generated an expanding body of evidence, particularly within GnRH antagonist protocols. In a meta-analysis including 10 RCTs (825 dual trigger, 813 hCG), the dual trigger has been reported with a significant increase in clinical pregnancy rate, live birth rate, and the number of mature oocytes, with the strongest benefit observed in fresh embryo transfer cycles, but not in frozen–thawed transfers [6]. Sloth et al. [7] reported an advantage in poor responders, reporting an increased likelihood of live birth (OR 2.65) compared with hCG alone. Conversely, a large RCT in 2474 poor responders (POSEIDON groups 3/4) reported significantly lower live birth rates in the dual trigger group compared with hCG (19% vs. 39%), raising concerns regarding the efficacy of dual trigger in this subgroup [8]. However, a closer look at this study shows that the LBR in the POR triggered by hCG was higher than that of the corresponding normal responder, figures indicating a “biased” control group with unacceptable high LBR. Other studies, however, have documented improved MII yield, embryo quality, and clinical pregnancy rates with dual trigger in poor responders, underscoring the heterogeneity of results [110]. Among normo-responders undergoing fresh ET cycles, the dual trigger appears particularly advantageous particularly for oocyte maturity-related outcomes. Although not directly evaluating trigger strategies, a meta-analysis by Liu et al., including 52 studies and 9950 participants, demonstrated equivalent live birth rates between GnRH agonist and antagonist protocols, but with a reduced risk of OHSS in antagonist cycles, highlighting the strategic importance of trigger choice in this context [89]. Alternative luteal support strategies with low-dose hCG have also been evaluated, showing comparable reproductive outcomes but with a tendency toward reduced OHSS when hCG was administered at the time of trigger [111]. A comparative summary of the main trigger protocols is reported in Table 2.

In parallel, Jayasena et al. [14] and Abbara et al. [15] investigated kisspeptin as a trigger in women at high risk of OHSS, confirming its safety and efficacy with both single- and double-dose regimens. A Cochrane review further demonstrated equivalence between recombinant and urinary hCG, with practical advantages for recombinant formulations [113]. Engmann et al., and Humaidan et al. [114,115] showed that hCG dose influences luteal phase progesterone profiles, and that sequential luteal support with hCG following GnRHa trigger achieves more stable progesterone levels. Seikkula et al. [116] reported that repeated administration of GnRHa for luteal support improves both clinical pregnancy and live birth rates. Evidence also suggests that dual trigger may increase the number of high-quality embryos and pregnancy rates after FET in women aged ≥35 years [117], while another study confirmed higher blastocyst yield and live birth rates compared with hCG alone [12]. Two additional meta-analyses reported a potential the benefit of dual trigger in normo-responders [118,119]. Alternative strategies include adding FSH to hCG as a trigger, which demonstrated non-inferiority in terms of oocyte competence and pregnancy rates [120], as well as evaluating different GnRHa dosages in oocyte donors, which showed no substantial differences in oocyte competence clinical pregnancy and live-birth [4]. Comparisons between freeze-all and fresh transfer following GnRHa plus low-dose hCG trigger have reported similar results, with OHSS occurring only in fresh transfer cycles [121]. Overall, multiple studies suggest that the dual trigger may improve MII yield, embryo quality, and selected clinical outcomes, although contradictory findings, particularly in poor responders, underscore the need for well-powered randomized trials [5,13,112,122,123,124]. Key clinical studies evaluating trigger strategies and reproductive outcomes are summarized in Table 3. Buhbut et al. [123] also reported encouraging results with intranasal GnRHa as luteal support following hCG trigger. The specific performance of the dual trigger in poor responders compared with hCG alone is detailed in Table 4. Taken together, the evidence on dual and combined triggers is promising but remains inconsistent. Randomized trials show mixed results, particularly in poor responders, and many supportive studies are observational or limited by sample size. Therefore, dual trigger strategies should be interpreted in light of the heterogeneity of the available evidence and applied selectively based on patient profile and treatment objectives.

**Figure 3:**
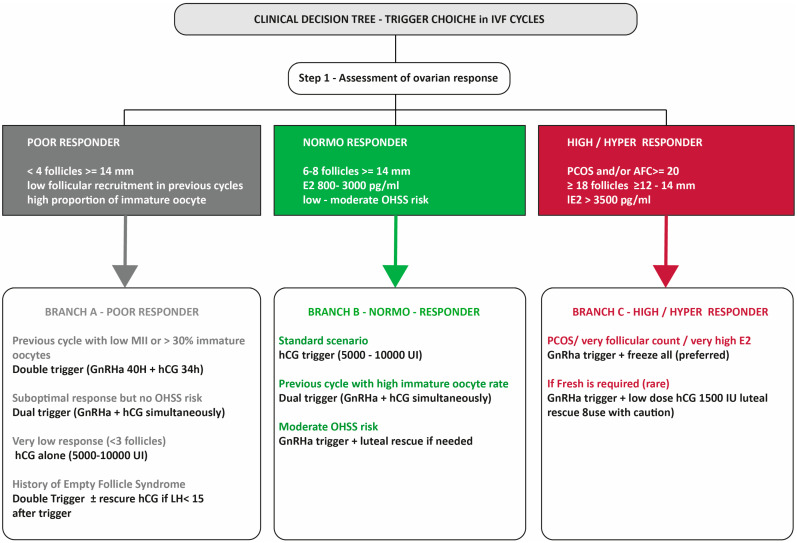
Clinical decision tree.

On the basis of the available evidence and the responder categories most frequently described in the literature, we developed a pragmatic clinical decision tree for trigger selection in IVF cycles (Figure 3). The scheme stratifies patients into poor, normo, and high or hyper responders according to follicle number, estradiol concentrations, and antral follicle count, and then illustrates possible trigger options within each branch (standard hCG trigger, GnRHa trigger with or without luteal rescue, dual trigger, or double trigger). This algorithm is intended as a conceptual aid that translates the heterogeneous data into a structured framework for daily practice rather than as a prescriptive guideline, and it should be adapted to local protocols and individual patient characteristics.

## 6. Conclusions

**Figure 4:**
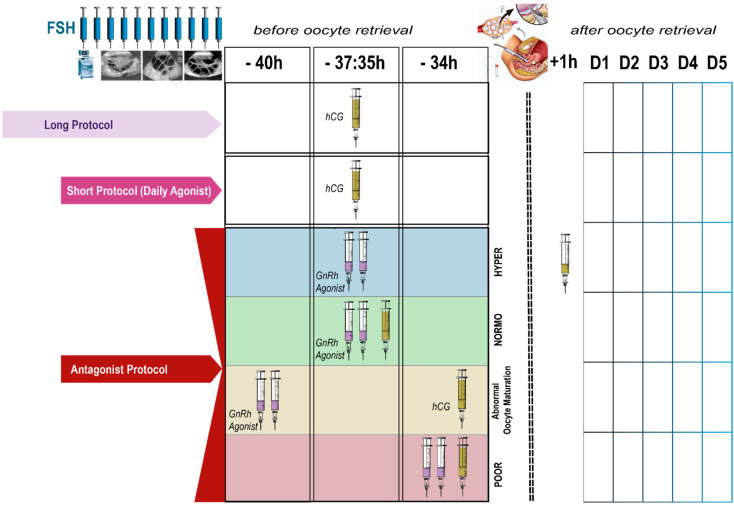
Summary of trigger strategies and clinical outcomes in IVF.

The choice of ovulation trigger for final oocyte maturation is a key determinant of both efficacy and safety in ART cycles. High-level evidence indicates that hCG reliably induces oocyte maturation but is associated with a significant risk of OHSS, particularly in high responders. In contrast, GnRHa triggering within GnRH-antagonist protocols almost abolishes severe OHSS, at the expense of a shorter and more fragile luteal phase that requires either segmentation (freeze-all) or intensive luteal support. Recent meta-analyses of randomized trials suggest that a dual trigger may improve oocyte maturity and, in some settings, live birth rates compared with hCG alone; however, these benefits are not universal, and at least one large RCT in poor responders has reported inferior live birth rates with dual trigger. Taken together, these data underscore that dual and double trigger protocols should be applied selectively, rather than as a default strategy. Importantly, the strongest data supporting kisspeptin as a trigger derive from early-phase trials in women at high risk of OHSS, showing high oocyte maturation rates and an excellent safety profile but still limited information on live birth and long-term outcomes. At present, kisspeptin should therefore be considered an emerging, predominantly research-based option rather than a routine clinical tool. Final oocyte maturation should be individualized according to OHSS risk, ovarian response, previous cycle performance, and patient priorities. Robust randomized trials, powered for live birth and stratified by responder profile, are urgently needed to refine timing, dose, and patient selection. Until such data are available, clinicians should prioritize safety, transparently communicate the level and quality of evidence supporting each strategy, and avoid over-generalizing results derived from highly selected populations to broader clinical practice. Figure 4 illustrates the main stimulation approaches used in IVF and shows how the choice of trigger is positioned within each protocol. It presents the long agonist approach, the short agonist approach and the antagonist approach, highlighting their timelines and the moment at which follicular monitoring and final oocyte maturation occur. The graphic also displays the types of medications applied for maturation, including agonist and hCG preparations, and visually organizes their use in relation to the different stimulation schemes. The layout offers a clear overview of how the trigger step is integrated into clinical practice across various stimulation strategies.

## Figures and Tables

**Table 1:** Summary of key clinical studies and meta-analyses included in this review.

First Author (Year)	Study Type/Level of Evidence	Population/Setting	Trigger Comparison	Main Outcomes
Youssef (2014) [1]	Cochrane meta-analyses (Level I)	IVF/ICSI cycles, GnRH-ant	Recombinant hCG vs. urinary hCG	No significant differences in ongoing pregnancy/live birth or OHSS risk between rhCG and uhCG.
Griesinger (2006) [2]	Systematic review and meta-analysis (Level I)	GnRH-ant cycles	GnRHa trigger vs. hCG	GnRHa markedly reduces/abolishes OHSS but may reduce clinical pregnancy/live birth without adequate luteal support.
Humaidan et al. (2013) [3]	RCTs (Level II)	High responders, GnRH-ant	GnRHa trigger ± individualized low-dose hCG	Adequate MII yield; improved luteal stability with on-demand low-dose hCG; markedly reduced OHSS risk.
Engmann et al. (2008) [4]	RCTs (Level II)	High responders/PCOS	GnRHa trigger + variable hCG rescue vs. hCG trigger	Comparable pregnancy rates with tailored luteal support; essentially no severe OHSS in GnRHa-based protocols.
Hu et al. (2021) [5]	Systematic review and meta-analysis (Level I)	GnRH-ant cycles	Dual trigger vs. hCG	Dual trigger associated with higher MII rate and improved clinical outcomes in selected populations; notable heterogeneity and small RCTs.
Hsia et al. (2023) [6]	Systematic review and meta-analysis of RCTs (Level I)	10 RCTs, 825 dual vs. 813 hCG	Dual trigger vs. hCG	Dual trigger increased retrieved oocytes (+1), MII (+0.8), CPR (OR 1.48), LBR (OR 1.61); no clear difference in OHSS.
Sloth et al. (2022) [7]	Systematic review and meta-analysis (Level I)	Poor responders (Bologna/POSEIDON)	Dual trigger vs. hCG	Higher MII yield; inconsistent effects on pregnancy/live birth; high heterogeneity.
Keskin et al. (2023) [8]	Multicentre RCT (Level II)	POSEIDON 3/4 (poor responders)	Dual trigger vs. hCG	Lower live birth in dual trigger group (19% vs. 39%); raises concern about use in POR.
Haas et al. (2014, 2016, 2019, 2020) [9,10,11,12]	RCT + pilot RCTs (Level II)	Normo-responders and low responders	Double/dual trigger vs. hCG	Increased MII and top-quality embryos; mixed effects on pregnancy/live birth; benefit appears subgroup-specific.
Zhu et al. (2021) [13]	Large retrospective cohort (Level III)	4438 freeze-all cycles	Dual trigger vs. hCG	Higher cumulative pregnancy and live birth with dual trigger in freeze-all strategy.
Jayasena et al. (2014) [14]	Phase II open-label study (Level II)	53 women undergoing IVF	Kisspeptin-54 single dose	Dose-dependent LH surge and oocyte maturation; CPR 23%; LBR limited by small sample.
Abbara et al. (2015) [15] Owens et al. (2018) [16]	Phase II randomized dose-finding trials (Level II)	High OHSS-risk patients	Kisspeptin-54 single vs. double dose	Oocyte maturation in 95%; ≥60% MII in ~70% with optimized dosing; CPR ~53%, LBR ~45%; no moderate/severe OHSS.
De Cesare et al. (2020) [17]	Large retrospective cohort (Level III)	>8000 IVF/ICSI cycles	hCG-trigger progesterone level	P4 ≥ 1.75 ng/mL at trigger associated with reduced CPR and LBR; supports freeze-all in this context.

**Table 2:** Comparison of trigger protocols in IVF/ICSI cycles.

Trigger Strategy	Retrieved Oocytes	MII Oocytes	Clinical Pregnancy (%)	Live Birth (%)	OHSS (%)
Urinary hCG (historical reference)	Typical yield; depends on response	~70–80% MII	~35–40% (variable)	~30–35%	~5–10%
Recombinant hCG (Cochrane review; equivalence to u-hCG)	Equivalent to urinary hCG	Equivalent	OR 1.15 (95% CI 0.89–1.49) vs. u-hCG [56]	Equivalent	Equivalent
GnRH agonist trigger (RCTs)	Similar or slightly lower	Variable (depends on luteal support)	Risk of reduced CPR without adequate luteal support	Equivalent with luteal reinforcement	Markedly reduced vs. hCG (RR ↓)
Dual trigger (simultaneous GnRHa + hCG) (meta-analyses; RCTs)	+1.05 oocyte retrieved vs. [6]	+0.8 0.82 MII vs. hCG (MD 0.82, 95% CI 0.48–1.16) [6]	OR 1.48 (95% CI 1.08–2.01) [6]	OR 1.61 (95% CI 1.16–2.25) [6]	Similarly to hCG; evidence heterogeneous
Double trigger (staggered GnRHa → hCG) (pilot studies; small cohorts)	Limited data	Limited data	Trend toward improvement [102,103,106]	Similar	Trend toward OHSS reduction [102,103,106]
Kisspeptin (early-phase clinical trials)	Comparable to GnRHa/hCG [48,50]	71% achieving MII ≥ 60% [16]	~45% CPR [15]	~45% [15]	No moderate or severe OHSS
hCG + FSH trigger (RCT)	Non-inferior	Non-inferior	RR 0.91 (95% CI 0.83–1.00) [112]	Non-inferior	Limited data

CPR = clinical pregnancy rate; OR = odds ratio; RR = risk ratio; OHSS = ovarian hyperstimulation syndrome. Data derived from meta-analysis of 10 RCTs.

**Table 3:** Comparison of trigger protocols in IVF/ICSI cycles (selected studies).

Study (Year)	Design	Population	Intervention	Key Outcomes	OHSS
Maged et al., 2021 [110]	RCT	200 normo-responders	hCG vs. GnRHa	Similar MII rate and fertilization; higher progesterone support needed with GnRHa	No severe OHSS
Keskin et al., 2023 [8]	RCT	180 antagonist cycles	GnRHa vs. hCG	Higher MII rate with GnRHa; similar CPR	Reduced vs. hCG
Zhou et al., 2022 [117]	Retrospective cohort	412 patients	Dual trigger vs. hCG	↑ MII oocytes, ↑ blastocyst rate	Similar
Haas et al., 2020 [12]	RCT	150 low responders	Double trigger vs. hCG	↑ Oocyte yield (NS), ↑ MII	No difference
Lambalk et al., 2017 [74]	Meta-analysis	52 studies, 9950 cycles	Agonist vs. antagonist protocols	Equivalent LBR; lower OHSS in antagonist cycles	OHSS ↓
Abbara et al., 2015 [15]	Phase I/II trial	High-risk OHSS	Kisspeptin-54	95% oocyte maturation; optimal dose identified	No moderate/severe
Engmann et al., 2019 [114]	RCT	High responders	GnRHa + low-dose hCG rescue	Improved luteal stability	0% severe OHSS
Vuong et al., 2016 [76]	Randomized dose-finding study	Oocyte donors	0.1–0.5 mg GnRHa	No difference in oocyte competence	NR

RCT = randomized controlled trial; NR = not reported; MII = metaphase II; CPR = clinical pregnancy rate; LBR = live birth rate; OHSS = ovarian hyperstimulation syndrome; NS = not significant.

**Table 4:** Efficacy of dual trigger compared with hCG alone in poor responders.

Study (Year)	Design	POR Criteria	Intervention	Key Findings	Pregnancy/LBR	OHSS
Sloth et al., 2022 [7]	Systematic review and meta-analysis	Bologna/POSEIDON	Dual trigger vs. hCG	↑ MII oocytes; inconsistent embryo outcomes	OR 1.48 CPR; OR 1.61 LBR	Similar
Keskin et al., 2023 [8]	RCT	Bologna criteria	Double trigger vs. hCG	No difference in retrieved oocytes	CPR NR; LBR 18%	No severe OHSS
Haas et al., 2020 [12]	RCT	Low responders	Double trigger vs. hCG	↑ MII, ↑ fertilization rate	CPR ↑ (NS)	Similar
Zhou et al., 2022 [117]	Retrospective	POSEIDON 3/4	Dual trigger vs. hCG	↑ MII oocytes, ↑ usable embryos	CPR ↑ (significant)	Similar
Keskin et al., 2023 [8]	RCT	2474 POR	Dual trigger vs. hCG	Lower LBR in dual trigger group (19% vs. 39%)	LBR significantly lower	Similar

POR = poor ovarian responder; CPR = clinical pregnancy rate; LBR = live birth rate; NR = not reported; OR = odds ratio; NS = not significant.

## Data Availability

No new data were created or analyzed in this study. Data sharing is not applicable to this article.

## References

[1] Youssef M.A., Van der Veen F., Al-Inany H.G., Mochtar M.H., Griesinger G., Nagi Mohesen M., Aboulfoutouh I., van Wely M. (2014). Gonadotropin-releasing hormone agonist versus HCG for oocyte triggering in antagonist-assisted reproductive technology. Cochrane Database Syst. Rev..

[2] Griesinger G., Diedrich K., Devroey P., Kolibianakis E.M. (2006). GnRH agonist for triggering final oocyte maturation in the GnRH antagonist protocol: A systematic review and meta-analysis. Hum. Reprod. Update.

[3] Humaidan P., Bredkjaer H.E., Bungum L., Bungum M., Grøndahl M.L., Westergaard L., Andersen C.Y. (2005). GnRH agonist (buserelin) or hCG for ovulation induction in GnRH antagonist IVF/ICSI cycles: A prospective randomized study. Hum. Reprod..

[4] Engmann L., DiLuigi A., Schmidt D., Nulsen J., Maier D., Benadiva C. (2008). The use of gonadotropin-releasing hormone (GnRH) agonist to induce oocyte maturation after cotreatment with GnRH antagonist in high-risk patients undergoing in vitro fertilization prevents the risk of ovarian hyperstimulation syndrome: A prospective randomized controlled study. Fertil. Steril..

[5] Hu K.L., Wang S., Ye X., Zhang D., Hunt S. (2021). GnRH agonist and hCG (dual trigger) versus hCG trigger for follicular maturation: A systematic review and meta-analysis of randomized trials. Reprod. Biol. Endocrinol..

[6] Hsia L.H., Lee T.H., Lin Y.H., Huang Y.Y., Chang H.J., Liu Y.L. (2023). Dual trigger improves the pregnancy rate in fresh in vitro fertilization (IVF) cycles compared with the human chorionic gonadotropin (hCG) trigger: A systematic review and meta-analysis of randomized trials. J. Assist. Reprod. Genet..

[7] Sloth A., Kjølhede M., Sarmon K.G., Knudsen U.B. (2022). Effect of dual trigger on reproductive outcome in low responders: A systematic PRISMA review and meta-analysis. Gynecol. Endocrinol..

[8] Keskin M., Ecemiş T., Atik A., Yeğen P., Kalkan E., Yücel G.S. (2023). Cycle outcomes of dual trigger (GnRH agonist+ hCG) versus human chorionic gonadotropin trigger alone in POSEDION group 3-4 poor-responders and normo-responders: A prospective randomized study. J. Gynecol. Obstet. Hum. Reprod..

[9] Haas J., Ophir L., Barzilay E., Machtinger R., Yung Y., Orvieto R., Hourvitz A. (2016). Standard human chorionic gonadotropin versus double trigger for final oocyte maturation results in different granulosa cells gene expressions: A pilot study. Fertil. Steril..

[10] Haas J., Zilberberg E., Nahum R., Mor Sason A., Hourvitz A., Gat I., Orvieto R. (2019). Does double trigger (GnRH-agonist + hCG) improve outcome in poor responders undergoing IVF-ET cycle? A pilot study. Gynecol. Endocrinol..

[11] Haas J., Kedem A., Machtinger R., Dar S., Hourvitz A., Yerushalmi G., Orvieto R. (2014). *HCG* (1500IU) administration on day 3 after oocytes retrieval, following GnRH-agonist trigger for final follicular maturation, results in high sufficient mid luteal progesterone levels—A proof of concept. J. Ovarian Res..

[12] Haas J., Bassil R., Samara N., Zilberberg E., Mehta C., Orvieto R., Casper R.F. (2020). GnRH agonist and hCG (dual trigger) versus hCG trigger for final follicular maturation: A double-blinded, randomized controlled study. Hum. Reprod..

[13] Zhu H., Zhao C., Pan Y., Zhou H., Jin X., Xu W., Zhang S. (2021). Dual Trigger for Final Follicular Maturation Improves Cumulative Live-Birth Rate in Ovarian Stimulation for Freeze-All In Vitro Fertilization/Intracytoplasmic Sperm Injection Cycles. Front Endocrinol.

[14] Jayasena C.N., Abbara A., Comninos A.N., Nijher G.M., Christopoulos G., Narayanaswamy S., Izzi-Engbeaya C., Sridharan M., Mason A.J., Warwick J. (2014). Kisspeptin-54 triggers egg maturation in women undergoing in vitro fertilization. J. Clin. Invest..

[15] Abbara A., Jayasena C.N., Christopoulos G., Narayanaswamy S., Izzi-Engbeaya C., Nijher G.M.K., Comninos A.N., Peters D., Buckley A., Ratnasabapathy R. (2015). Efficacy of Kisspeptin-54 to Trigger Oocyte Maturation in Women at High Risk of Ovarian Hyperstimulation Syndrome (OHSS) During In Vitro Fertilization (IVF) Therapy. J. Clin. Endocrinol. Metab..

[16] Owens L.A., Abbara A., Lerner A., O’Floinn S., Christopoulos G., Khanjani S., Islam R., Hardy K., Hanyaloglu A.C., Lavery S.A. (2018). The direct and indirect effects of kisspeptin-54 on granulosa lutein cell function. Hum. Reprod..

[17] De Cesare R., Morenghi E., Cirillo F., Ronchetti C., Canevisio V., Persico P., Baggiani A., Sandri M.T., Levi-Setti P.E. (2020). The Role of hCG Triggering Progesterone Levels: A Real-World Retrospective Cohort Study of More Than 8000 IVF/ICSI Cycles. Front. Endocrinol..

[18] Durinzi K.L., Saniga E.M., Lanzendorf S.E. (1995). The relationship between size and maturation in vitro in the unstimulated human oocyte. Fertil. Steril..

[19] Rosen M., Shen S., Dobson A., Rinaudo P., McCulloch C., Cedars M. (2008). A quantitative assessment of follicle size on oocyte developmental competence. Fertil. Steril..

[20] Mohr-Sasson A., Orvieto R., Blumenfeld S., Axelrod M., Mor-Hadar D., Grin L., Aizer A., Haas J. (2020). The association between follicle size and oocyte development as a function of final follicular maturation triggering. Reprod. Biomed. Online.

[21] Campbell B.K., McNeilly A.S. (1996). Follicular dominance and oocyte maturation. Zygote.

[22] Driancourt M.A., Thuel B. (1998). Control of oocyte growth and maturation by follicular cells and molecules present in follicular fluid: A review. Reprod. Nutr. Dev..

[23] Hunter M.G. (1998). Follicular factors regulating oocyte maturation and quality. Hum. Fertil..

[24] Shapiro B.S., Daneshmand S.T., Garner F.C., Aguirre M., Hudson C. (2011). Comparison of “triggers” using leuprolide acetate alone or in combination with low-dose human chorionic gonadotropin. Fertil. Steril..

[25] Wittmaack F.M., Kreger D.O., Blasco L., Tureck R.W., Mastroianni L., Lessey B.A. (1994). Effect of follicular size on oocyte retrieval, fertilization, cleavage, and embryo quality in IVF cycles: A 6-year data collection. Fertil. Steril..

[26] Shmorgun D., Hughes E., Mohide P., Roberts R. (2010). Prospective cohort study of three- versus two-dimensional ultrasound for prediction of oocyte maturity. Fertil. Steril..

[27] Abbara A., Vuong L.N., Ho V.N.A., Clarke S.A., Jeffers L., Comninos A.N., Salim R., Ho T.M., Kelsey T.W., Trew G.H. (2018). Follicle size on day of trigger most likely to yield a mature oocyte. Front. Endocrinol..

[28] Helmer A., Magaton I., Stalder O., Stute P., Surbek D., von Wolff M. (2022). Optimal Timing of Ovulation Triggering to Achieve Highest Success Rates in Natural Cycles-An Analysis Based on Follicle Size and Oestradiol Concentration in Natural Cycle IVF. Front. Endocrinol..

[29] Yenigül N.N., Özelci R., Başer E., Dilbaz S., Aldemir O., Dilbaz B., Moraloğlu Tekin Ö. (2023). Does the decrease in E2 levels between the trigger of ovulation and embryo transfer affect the reproductive outcome in IVF-ICSI cycles?. Turk. J. Obstet. Gynecol..

[30] Orvieto RRabinson J., Meltcer S., Gemer O., Anteby E.Y., Zohav E. (2008). Does physicians’ experience influence in vitro fertilization success in patients undergoing controlled ovarian hyperstimulation with GnRH antagonists?. Fertil. Steril..

[31] Baldini D., Bartoli V.M., Mastrorocco A., Ferri D., Dellino M., Laganà A.S., Hatirnaz S., Baldini G.M., Malvasi A., Vimercati A. (2024). Progesterone peak influences embryonic developmental morphokinetics on trigger day? A retrospective study. J. Ovarian Res..

[32] Baldini D., Savoia M.V., Sciancalepore A.G., Malvasi A., Vizziello D., Beck R., Vizziello G. (2018). High progesterone levels on the day of hCG administration do not affect embryo quality and reproductive outcomes of frozen embryo transfers. Clin. Ter..

[33] Davar R., Naghshineh E., Neghab N. (2017). The effect of 24 hours delay in oocyte maturation triggering in IVF/ICSI cycles with antagonist protocol and not-elevated progesterone: A randomized control trial. Int. J. Reprod. Biomed..

[34] Vandekerckhove F., Gerris J., Vansteelandt S., De Baerdemaeker A., Tilleman K., De Sutter P. (2014). Delaying the oocyte maturation trigger by one day leads to a higher metaphase II oocyte yield in IVF/ICSI: A randomized controlled trial. Reprod. Biol. Endocrinol..

[35] Chen Q.L., Shuai J., Pei L., Huang G.N., Ye H. (2021). Impact of trigger timing of gonadotropin-releasing hormone antagonist regimen for infertility patients of various ages. Zhonghua Fu Chan Ke Za Zhi.

[36] Ranit H., Shmuel H., Ahlad A., Shirley G., Meny H., Tal I., Assaf B.-M., Chana A.L., Yaakov B., Efrat E.-B. (2024). Is there a preferred time interval between GnRH agonist trigger and oocyte retrieval in GnRH antagonist cycles? A retrospective cohort of planned fertility preservation cycles. J. Assist. Reprod. Genet..

[37] Weng Y.W., Lin M.H., Kuo-Kuang Lee R., Li S.H., Jou Q.B., Chen Y.J. (2022). Is there an optimal timing interval between hCG trigger and oocyte vitrification?. Taiwan. J. Obstet. Gynecol..

[38] Cadenas J., Adrados C.S., Kumar A., Kalra B., Salto Mamsen L., Andersen C.Y. (2025). Regulating human oocyte maturation in vitro: A hypothesis based on oocytes retrieved from small antral follicles during ovarian tissue cryopreservation. J. Assist. Reprod. Genet..

[39] Zelinski-Wooten M.B., Lanzendorf S.E., Wolf D.P., Chandrasekher Y.A., Stouffer R.L. (1991). Titrating luteinizing hormone surge requirements for ovulatory changes in primate follicles. I. Oocyte maturation and corpus luteum function. J. Clin. Endocrinol. Metab..

[40] Arroyo A., Kim B., Yeh J. (2020). Luteinizing Hormone Action in Human Oocyte Maturation and Quality: Signaling Pathways, Regulation, and Clinical Impact. Reprod. Sci..

[41] Zhang H., Lu S., Xu R., Tang Y., Liu J., Li C., Wei J., Yao R., Zhao X., Wei Q. (2020). Mechanisms of Estradiol-induced EGF-like Factor Expression and Oocyte Maturation via G Protein-coupled Estrogen Receptor. Endocrinology.

[42] Alak B.M., Smith G.D., Woodruff T.K., Stouffer R.L., Wolf D.P. (1996). Enhancement of primate oocyte maturation and fertilization in vitro by inhibin A and activin A. Fertil. Steril..

[43] Chang C.L., Wang T.H., Horng S.G., Wu H.M., Wang H.S., Soong Y.K. (2002). The concentration of inhibin B in follicular fluid: Relation to oocyte maturation and embryo development. Hum. Reprod..

[44] Lawrenz B., Depret Bixio L., Coughlan C., Andersen C.Y., Melado L., Kalra B., Savjani G., Fatemi H.M., Kumar A. (2020). Inhibin A: A promising predictive parameter for determination of final oocyte maturation in ovarian stimulation for IVF/ICSI. Front. Endocrinol..

[45] Kala M., Shaikh M.V., Nivsarkar M. (2016). Equilibrium between anti-oxidants and reactive oxygen species: A requisite for oocyte development and maturation. Reprod. Med. Biol..

[46] Masumi S., Lee E.B., Dilower I., Upadhyaya S., Chakravarthi V.P., Fields P.E., Rumi M.A.K. (2022). The role of kisspeptin signaling in oocyte maturation. Front. Endocrinol..

[47] Kuspinar G., Cakır C., Kasapoglu I., Saribal S., Oral B., Budak F., Uncu G., Avcı B. (2022). The Kisspeptin and Kisspeptin receptor in follicular microenvironment: Is that really necessary for oocyte maturation and fertilisation?. J. Obstet. Gynaecol..

[48] Sharma B., Koysombat K., Comninos A.N., Dhillo W.S., Abbara A. (2022). Use of kisspeptin to trigger oocyte maturation during *in vitro* fertilisation (IVF) treatment. Front. Endocrinol..

[49] Taniguchi Y., Kuwahara A., Tachibana A., Yano Y., Yano K., Yamamoto Y., Yamasaki M., Iwasa T., Hinokio K., Matsuzaki T. (2017). Intra-follicular kisspeptin levels are related to oocyte maturation and gonadal hormones in patients undergoing ART. Reprod. Med. Biol..

[50] Kasum M., Franulić D., Čehić E., Orešković S., Lila A., Ejubović E. (2017). Kisspeptin as a promising oocyte maturation trigger for IVF in humans. Gynecol. Endocrinol..

[51] Singh N., Girish B., Malhotra N., Mahey R., Perumal V. (2019). Does Double Dose of Recombinant Human Chorionic Gonadotropin for Final Follicular Maturation in In vitro Fertilization Cycles Improve Oocyte Quality: A Prospective Randomized Study. J. Hum. Reprod. Sci..

[52] Kolibianakis E.M., Papanikolaou E.G., Tournaye H., Camus M., Van Steirteghem A.C., Devroey P. (2007). Triggering final oocyte maturation using different doses of human chorionic gonadotropin: A randomized pilot study in patients with polycystic ovary syndrome treated with gonadotropin-releasing hormone antagonists and recombinant follicle-stimulating hormone. Fertil. Steril..

[53] Lin H., Wang W., Li Y., Chen X., Yang D., Zhang Q. (2011). Triggering final oocyte maturation with reduced doses of hCG in IVF/ICSI: A prospective, randomized and controlled study. Eur. J. Obstet. Gynecol. Reprod. Biol..

[54] Ashkenazi J., Bar-Hava I., Meltcer S., Rabinson J., Anteby E.Y., Orvieto R. (2009). The possible influence of increased body mass index on the clinical efficacy of standard human chorionic gonadotropin dosage. Clin. Exp. Obstet. Gynecol..

[55] Svenstrup L., Möller S., Fedder J., Pedersen D.E., Erb K., Andersen C.Y., Humaidan P. (2022). Does the HCG trigger dose used for IVF impact luteal progesterone concentrations? a randomized controlled trial. Reprod. Biomed. Online.

[56] Youssef M.A., Al-Inany H.G., Aboulghar M., Mansour R., Abou-Setta A.M. (2011). Recombinant versus urinary human chorionic gonadotrophin for final oocyte maturation triggering in IVF and ICSI cycles. Cochrane Database Syst. Rev..

[57] Andersen C.Y., Kelsey T., Mamsen L.S., Vuong L.N. (2020). Shortcomings of an unphysiological triggering of oocyte maturation using human chorionic gonadotropin. Fertil. Steril..

[58] Kol S., Humaidan P. (2013). GnRH agonist triggering: Recent developments. Reprod. Biomed. Online.

[59] Orvieto R., Nahum R., Frei J., Zandman O., Frenkel Y., Haas J. (2021). GnRH-Agonist Ovulation Trigger in Patients Undergoing Controlled Ovarian Hyperstimulation for IVF with Stop GnRH-Agonist Combined with Multidose GnRH-Antagonist Protocol. Gynecol. Obstet. Invest..

[60] Budinetz T.H., Mann J.S., Griffin D.W., Benadiva C.A., Nulsen J.C., Engmann L.L. (2014). Maternal and neonatal outcomes after gonadotropin-releasing hormone agonist trigger for final oocyte maturation in patients undergoing in vitro fertilization. Fertil. Steril..

[61] Hoff J.D., Quigley M.E., Yen S.S. (1983). Hormonal dynamics at midcycle: A reevaluation. J. Clin. Endocrinol. Metab..

[62] Babayof R., Margalioth E.J., Huleihel M., Amash A., Zylber-Haran E., Gal M., Brooks B., Mimoni T., Eldar-Geva T. (2006). Serum inhibin A, VEGF and TNFalpha levels after triggering oocyte maturation with GnRH agonist compared with HCG in women with polycystic ovaries undergoing IVF treatment: A prospective randomized trial. Hum. Reprod..

[63] Engmann L., DiLuigi A., Schmidt D., Benadiva C., Maier D., Nulsen J. (2008). The effect of luteal phase vaginal estradiol supplementation on the success of in vitro fertilization treatment: A prospective randomized study. Fertil. Steril..

[64] Garcia-Velasco J.A., Motta L., López A., Mayoral M., Cerrillo M., Pacheco A. (2010). Low-dose human chorionic gonadotropin versus estradiol/progesterone luteal phase support in gonadotropin-releasing hormone agonist-triggered assisted reproductive technique cycles: Understanding a new approach. Fertil. Steril..

[65] DiLuigi A.J., Engmann L., Schmidt D.W., Maier D.B., Nulsen J.C., Benadiva C.A. (2010). Gonadotropin-releasing hormone agonist to induce final oocyte maturation prevents the development of ovarian hyperstimulation syndrome in high-risk patients and leads to improved clinical outcomes compared with coasting. Fertil. Steril..

[66] Kol S., Muchtar M. (2005). Recombinant gonadotrophin-based, ovarian hyperstimulation syndrome-free stimulation of the high responder: Suggested protocol for further research. Reprod. Biomed. Online.

[67] Humaidan P., Kol S., Papanikolaou E., Copenhagen GnRH Agonist Triggering Workshop Group (2011). GnRH agonist for triggering final oocyte maturation: Time for a change of practice?. Hum. Reprod. Update.

[68] Seyhan A., Ata B., Polat M., Son W.Y., Yarali H., Dahan M.H. (2013). Severe early ovarian hyperstimulation syndrome following GnRH agonist trigger with the addition of 1500 IU hCG. Hum. Reprod..

[69] Ling L.P., Phoon J.W., Lau M.S., Chan J.K., Viardot-Foucault V., Tan T.Y., Nadarajah S., Tan H.H. (2014). GnRH agonist trigger and ovarian hyperstimulation syndrome: Relook at ‘freeze-all strategy’. Reprod. Biomed. Online.

[70] Orvieto R., Vanni V.S. (2017). Ovarian hyperstimulation syndrome following GnRH agonist trigger- think ectopic. J. Assist. Reprod. Genet..

[71] Al-Inany H.G., Youssef M.A., Ayeleke R.O., Brown J., Lam W.S., Broekmans F.J. (2016). Gonadotrophin-releasing hormone antagonists for assisted reproductive technology. Cochrane Database Syst. Rev..

[72] Jing M., Lin C., Zhu W., Tu X., Chen Q., Wang X., Zheng Y., Zhang R. (2020). Cost-effectiveness analysis of GnRH-agonist long-protocol and GnRH-antagonist protocol for in vitro fertilization. Sci. Rep..

[73] Murber Á., Fancsovits P., Ledó N., Gilán Z.T., Rigó J., Urbancsek J. (2009). Impact of GnRH analogues on oocyte/embryo quality and embryo development in in vitro fertilization/intracytoplasmic sperm injection cycles: A case control study. Reprod. Biol. Endocrinol..

[74] Lambalk C.B., Banga F.R., Huirne J.A., Toftager M., Pinborg A., Homburg R., van der Veen F., van Wely M. (2017). GnRH antagonist versus long agonist protocols in IVF: Systematic review and meta-analysis accounting for patient type. Hum. Reprod. Update.

[75] Humaidan P., Engmann L., Benadiva C. (2015). Luteal phase supplementation after gonadotropin-releasing hormone agonist trigger in fresh ET: The American versus European approaches. Fertil. Steril..

[76] Vuong T.N., Ho M.T., Ha T.D., Phung H.T., Huynh G.B., Humaidan P. (2016). Gonadotropin-releasing hormone agonist trigger in oocyte donors co-treated with a gonadotropin-releasing hormone antagonist: A dose-finding study. Fertil. Steril..

[77] Andersen C.Y., Humaidan P., Ejdrup H.B., Bungum L., Grøndahl M.L., Westergaard L.G. (2006). Hormonal characteristics of follicular fluid from women receiving either GnRH agonist or hCG for ovulation induction. Hum. Reprod..

[78] Kummer N.E., Feinn R.S., Griffin D.W., Nulsen J.C., Benadiva C.A., Engmann L.L. (2013). Predicting successful induction of oocyte maturation after GnRH agonist trigger. Hum. Reprod..

[79] Chang F.E., Beall S.A., Cox J.M., Richter K.S., DeCherney A.H., Levy M.J. (2016). Assessing the adequacy of gonadotropin-releasing hormone agonist leuprolide to trigger oocyte maturation and management of inadequate response. Fertil. Steril..

[80] O’Neill K.E., Senapati S., Dokras A. (2015). Use of gonadotropin-releasing hormone agonist trigger during in vitro fertilization is associated with similar endocrine profiles and oocyte measures in women with and without polycystic ovary syndrome. Fertil. Steril..

[81] El Tokhy O., Kopeika J., El-Toukhy T. (2016). An update on the prevention of ovarian hyperstimulation syndrome. Womens Health.

[82] Roque M., Haahr T., Geber S., Esteves S.C., Humaidan P. (2019). Fresh versus elective frozen embryo transfer in IVF/ICSI cycles: A systematic review and meta-analysis of reproductive outcomes. Hum. Reprod. Update.

[83] Stormlund S., Løssl K., Zedeler A., Bogstad J., Prætorius L., Nielsen H.S., Bungum M., Skouby S.O., Mikkelsen A.L., Andersen A.N. (2017). Comparison of a ‘freeze-all’ strategy including GnRH agonist trigger versus a ‘fresh transfer’ strategy including hCG trigger in assisted reproductive technology (ART): A study protocol for a randomised controlled trial. BMJ Open.

[84] Wiser A., Klement A.H., Shavit T., Berkovitz A., Koren R.R., Gonen O., Amichay K., Shulman A. (2019). Repeated GnRH agonist doses for luteal support: A proof of concept. Reprod. Biomed. Online.

[85] Orvieto R., Rabinson J., Meltzer S., Zohav E., Anteby E., Homburg R. (2006). Substituting hCG with GnRH agonist to trigger final follicular maturation- a retrospective comparison of three different ovarian stimulation protocols. Reprod. Biomed. Online.

[86] Humaidan P., Papanikolaou E.G., Kyrou D., Alsbjerg B., Polyzos N.P., Devroey P., Fatemi H.M. (2012). The luteal phase after GnRH-agonist triggering of ovulation: Present and future perspectives. Reprod. Biomed. Online.

[87] Yanushpolsky E.H. (2015). Luteal phase support in in vitro fertilization. Semin. Reprod. Med..

[88] Lawrenz B., Humaidan P., Kol S., Fatemi H.M. (2018). GnRHa trigger and luteal coasting: A new approach for the ovarian hyperstimulation syndrome high-risk patient?. Reprod. Biomed. Online.

[89] Liu Y., Huang K., Chen C., Wen L., Lei M., Guo Y., Tang B. (2023). Effect of luteal-phase GnRH agonist on frozen-thawed embryo transfer during artificial cycles: A randomised clinical pilot study. Front. Endocrinol..

[90] Orvieto R. (2015). Triggering final follicular maturation—hCG, GnRH-agonist or both, when and to whom?. J. Ovarian Res..

[91] Popovic-Todorovic B., Santos-Ribeiro S., Drakopoulos P., De Vos M., Racca A., Mackens S., Thorrez Y., Verheyen G., Tournaye H., Quintero L. (2019). Predicting suboptimal oocyte yield following GnRH agonist trigger by measuring serum LH at the start of ovarian stimulation. Hum. Reprod..

[92] Hershko Klement A., Orvieto R., Esh Broder E., Frei J., Solnica A., Zandman O., Holzer H., Haas J. (2021). How far is too far? Does time interval between GnRH antagonist and GnRH agonist trigger in GnRH antagonist cycles matter?. Reprod. Biomed. Online.

[93] Humaidan P., Papanikolaou E.G., Tarlatzis B.C. (2009). GnRHa to trigger final oocyte maturation: Time to reconsider. Hum. Reprod..

[94] Horowitz E., Mizrachi Y., Farhi J., Raziel A., Weissman A. (2020). Does the interval between last GnRH antagonist dose and GnRH agonist trigger affect oocyte recovery and maturation rates?. Reprod. Biomed. Online.

[95] Reissmann T., Schally A.V., Bouchard P., Riethmiiller H., Engel J.B. (2000). The LHRH antagonist cetrorelix: A review. Hum. Reprod. Update.

[96] Zaat T., Zagers M., Mol F., Goddijn M., van Wely M., Mastenbroek S. (2021). Fresh versus frozen embryo transfers in assisted reproduction. Cochrane Database Syst. Rev..

[97] Toftager M., Bogstad J., Bryndorf T., Løssl K., Roskær J., Holland T., Prætorius L., Zedeler A., Nilas L., Pinborg A. (2016). Risk of severe ovarian hyperstimulation syndrome in GnRH antagonist versus GnRH agonist protocol: RCT including 1050 first IVF/ICSI cycles. Hum. Reprod..

[98] Rahav Koren R., Miller N., Moran R., Decter D., Berkowitz A., Haikin Herzberger E., Wiser A. (2022). GnRH agonist-triggering ovulation in women with advanced age. Sci. Rep..

[99] Orvieto R. (2012). Intensive luteal-phase support with oestradiol and progesterone after GnRH-agonist triggering: Does it help?. Reprod. Biomed. Online.

[100] Bodri D., Guillén J.J., Trullenque M., Schwenn K., Esteve C., Coll O. (2010). Early ovarian hyperstimulation syndrome is completely prevented by gonadotropin releasing-hormone agonist triggering in high-risk oocyte donor cycles: A prospective, luteal-phase follow-up study. Fertil. Steril..

[101] Shapiro B.S., Daneshmand S.T., Garner F.C., Aguirre M., Thomas S. (2008). Gonadotropin-releasing hormone agonist combined with a reduced dose of human chorionic gonadotropin for final oocyte maturation in fresh autologous cycles of in vitro fertilization. Fertil. Steril..

[102] Cerrillo M., Rodríguez S., Mayoral M., Pacheco A., Martínez-Salazar J., Garcia-Velasco J.A. (2009). Differential regulation of VEGF after final oocyte maturation with GnRH agonist versus hCG: A rationale for OHSS reduction. Fertil. Steril..

[103] Decleer W., Osmanagaoglu K., Seynhave B., Kolibianakis S., Tarlatzis B., Devroey P. (2014). Comparison of hCG triggering versus hCG in combination with a GnRH agonist: A prospective randomized controlled trial. Facts Views Vis. Obgyn.

[104] Zilberberg E., Haas J., Dar S., Kedem A., Machtinger R., Orvieto R. (2015). Co-administration of GnRH-agonist and hCG, for final oocyte maturation (double trigger), in patients with low proportion of mature oocytes. Gynecol. Endocrinol..

[105] Beck-Fruchter R., Weiss A., Lavee M., Geslevich Y., Shalev E. (2012). Empty follicle syndrome: Successful treatment in a recurrent case and review of the literature. Hum. Reprod..

[106] Lok F., Pritchard J., Lashen H. (2003). Successful treatment of empty follicle syndrome by triggering endogenous LH surge using GnRH agonist in an antagonist down-regulated IVF cycle. Hum. Reprod..

[107] Humaidan P., Ejdrup Bredkjær H., Westergaard L.G., Yding Andersen C. (2010). 1,500 IU human chorionic gonadotropin administered at oocyte retrieval rescues the luteal phase when gonadotropin-releasing hormone agonist is used for ovulation induction: A prospective, randomized, controlled study. Fertil. Steril..

[108] Papanikolaou E.G., Verpoest W., Fatemi H., Tarlatzis B., Devroey P., Tournaye H. (2011). A novel method of luteal supplementation with recombinant luteinizing hormone when a gonadotropin-releasing hormone agonist is used instead of human chorionic gonadotropin for ovulation triggering: A randomized prospective proof of concept study. Fertil. Steril..

[109] Tu B., Zhang H., Chen L., Yang R., Liu P., Li R., Qiao J. (2024). Co-administration of GnRH-agonist and hCG (double trigger) for final oocyte maturation increases the number of top-quality embryos in patients undergoing IVF/ICSI cycles. J. Ovarian Res..

[110] Maged A.M., Ragab M.A., Shohayeb A., Saber W., Ekladious S., Hussein E.A., El-Mazny A., Hany A. (2021). Comparative study between single versus dual trigger for poor responders in GnRH-antagonist ICSI cycles: A randomized controlled study. Int. J. Gynaecol. Obstet..

[111] Benadiva C., Engmann L. (2018). Luteal phase support after gonadotropin-releasing hormone agonist triggering: Does it still matter?. Fertil. Steril..

[112] Ansaripour S., Tamizi N., Sadeghi M.R., Mohammad-Akbari A. (2022). Comparison of Triggering Final Oocyte Maturation with Follicle Stimulating Hormone Plus Human Chorionic Gonadotropin, versus Human Chorionic Gonadotropin Alonein Normoresponder Women Undergoing Intracytoplasmic Sperm Injection: A Randomized Clinical Trial. Int. J. Fertil. Steril..

[113] Youssef M.A., Abdelmoty H.I., Ahmed M.A., Elmohamady M. (2015). GnRH agonist for final oocyte maturation in GnRH antagonist co-treated IVF/ICSI treatment cycles: Systematic review and meta-analysis. J. Adv. Res..

[114] Engmann L.L., Maslow B.S., Kaye L.A., Griffin D.W., DiLuigi A.J., Schmidt D.W., Grow D.R., Nulsen J.C., Benadiva C.A. (2019). Low dose human chorionic gonadotropin administration at the time of gonadotropin releasing-hormone agonist trigger versus 35 h later in women at high risk of developing ovarian hyperstimulation syndrome—A prospective randomized double-blind clinical trial. J. Ovarian Res..

[115] Humaidan P., Polyzos N.P., Alsbjerg B., Erb K., Mikkelsen A.L., Elbaek H.O., Papanikolaou E.G., Andersen C.Y. (2013). GnRHa trigger and individualized luteal phase hCG support according to ovarian response to stimulation: Two prospective randomized controlled multi-centre studies in IVF patients. Hum. Reprod..

[116] Seikkula J., Ahinko K., Polo-Kantola P., Anttila L., Hurme S., Tinkanen H., Jokimaa V. (2018). Mid-luteal phase gonadotropin-releasing hormone agonist support in frozen-thawed embryo transfers during artificial cycles: A prospective interventional pilot study. J. Gynecol. Obstet. Hum. Reprod..

[117] Zhou C., Yang X., Wang Y., Xi J., Pan H., Wang M., Zhou Y., Xiao Y. (2022). Ovulation triggering with hCG alone, GnRH agonist alone or in combination? A randomized controlled trial in advanced-age women undergoing IVF/ICSI cycles. Hum. Reprod..

[118] He F.F., Hu W., Yong L., Li Y.M. (2023). Triggering of ovulation for GnRH-antagonist cycles in normal and low ovarian responders undergoing IVF/ICSI: A systematic review and meta-analysis of randomized trials. Eur. J. Obstet. Gynecol. Reprod. Biol..

[119] Santos-Ribeiro S., Mackens S., Popovic-Todorovic B., Racca A., Polyzos N.P., Van Landuyt L., Drakopoulos P., de Vos M., Tournaye H., Blockeel C. (2020). The freeze-all strategy versus agonist triggering with low-dose hCG for luteal phase support in IVF/ICSI for high responders: A randomized controlled trial. Hum. Reprod..

[120] Dong L., Lian F., Wu H., Xiang S., Li Y., Wei C., Yu X., Xin X. (2022). Reproductive outcomes of dual trigger with combination GnRH agonist and hCG versus trigger with hCG alone in women undergoing IVF/ICSI cycles: A retrospective cohort study with propensity score matching. BMC Pregnancy Childbirth.

[121] Chen K., Zhang C., Chen L., Zhao Y., Li H. (2024). Reproductive outcomes of dual trigger therapy with GnRH agonist and hCG versus hCG trigger in women with diminished ovarian reserve: A retrospective study. Reprod. Biol. Endocrinol..

[122] González V.G., Triana A.M., García I.S., Nieto S.O., Urrutia M.C., García I.C., Gastañaga-Holguera T. (2023). Dual trigger vs. Conventional trigger outcomes in In Vitro Fertilization. Systematic review and meta-analysis. JBRA Assist. Reprod..

[123] Buhbut E., Nabulsi R., Avigdor G., Ben-Ami I. (2023). Comparison of pregnancy rates in antagonist cycles after luteal support with GnRH-agonist versus progesterone: Prospective randomized study. Arch. Gynecol. Obstet..

[124] Lin M.H., Wu F.S., Lee R.K., Li S.H., Lin S.Y., Hwu Y.M. (2013). Dual trigger with combination of gonadotropin-releasing hormone agonist and human chorionic gonadotropin significantly improves the live-birth rate for normal responders in GnRH-antagonist cycles. Fertil. Steril..

